# Terra Nostra Garden 2023 dataset of *Camellia* species, hybrids and their cultivars (Azores, Portugal)

**DOI:** 10.3897/BDJ.11.e109193

**Published:** 2023-08-09

**Authors:** Carina Costa, Fernando Costa, António Trota, Paulo A. V. Borges, Paulo Monjardino, Maria J. Pereira

**Affiliations:** 1 Terra Nostra Garden Hotel, Rua Padre José Jacinto Botelho 5, 9675-061, Furnas, Azores, Portugal Terra Nostra Garden Hotel, Rua Padre José Jacinto Botelho 5, 9675-061 Furnas, Azores Portugal; 2 CGeo -Centre of Geosciences (University of Coimbra) and University of the Azores - Campus de Ponta Delgada, Rua da Mãe de Deus, 9500-321, Ponta Delgada, Azores, Portugal CGeo -Centre of Geosciences (University of Coimbra) and University of the Azores - Campus de Ponta Delgada, Rua da Mãe de Deus, 9500-321 Ponta Delgada, Azores Portugal; 3 cE3c- Centre for Ecology, Evolution and Environmental Changes, Azorean Biodiversity Group, CHANGE – Global Change and Sustainability InstitutcE3c- Centre for Ecology, Evolution and Environmental Changes/Azorean Biodiversity Group, CHANGE – Global Change and Sustainability Institute, School of Agricultural and Environmental Sciences, University of the Azores, Rua Capitão João d´Ávila, Pico da Urze, 9700-042, Angra do Heroísmo, Azores, Portugal cE3c- Centre for Ecology, Evolution and Environmental Changes, Azorean Biodiversity Group, CHANGE – Global Change and Sustainability InstitutcE3c- Centre for Ecology, Evolution and Environmental Changes/Azorean Biodiversity Group, CHANGE – Global Change and Sustainability Institute, School of Agricultural and Environmental Sciences, University of the Azores, Rua Capitão João d´Ávila, Pico da Urze, 9700-042 Angra do Heroísmo, Azores Portugal; 4 CBA – Centre of Biotecnology of the Azores, University of the Azores - Campus de Angra do Heroísmo, Rua Capitão João d´Ávila, Pico da Urze, 9700-042, Angra do Heroísmo, Azores, Portugal CBA – Centre of Biotecnology of the Azores, University of the Azores - Campus de Angra do Heroísmo, Rua Capitão João d´Ávila, Pico da Urze, 9700-042 Angra do Heroísmo, Azores Portugal; 5 CBA – Centre of Biotecnology of the Azores, University of the Azores - Campus de Ponta Delgada, Rua da Mãe de Deus, 9500-321, Ponta Delgada, Azores, Portugal CBA – Centre of Biotecnology of the Azores, University of the Azores - Campus de Ponta Delgada, Rua da Mãe de Deus, 9500-321 Ponta Delgada, Azores Portugal

**Keywords:** Azores Archipelago, Camellia Collections, digital datase, Historical Gardens, 19^th^ century gardens, Romantic Gardens

## Abstract

**Background:**

Camellias cultivars collections, comprising an exquisite array of meticulously bred and curated camellia varieties, emerged as indispensable elements within the resplendent 19^th^ century gardens that adorned landscapes across the globe. The heart of Terra Nostra Garden, nestled within the captivating surroundings of the Furnas volcano on S. Miguel Island (Azores, Portugal), started in the year 1782 as an enchanting fishpond garden, strategically positioned in front of the summer house belonging to the esteemed Thomas Hickling, the American vice-consul. Soon this garden was enlarged and embellished with species from several continents. This tradition continued during all the 19^th^ century with the Viscount Duarte Borges da Câmara Medeiros (1848-1872) and his son the Marquis António Borges Medeiros Dias da Câmara e Sousa (1872-1913). In 1933, the 12 hectares property was acquired by the Terra Nostra Society, led by Vasco Bensaude and increased to 12.5 hectares. In 1935, the current Terra Nostra Garden Hotel was inaugurated and, two years later, the Terra Nostra Society reopened the botanical garden attached to the hotel, now called Terra Nostra Garden. Beginning in 1982, the Head Gardener embarked on a transformative journey, dedicating his expertise to the meticulous renovation and expansion of numerous botanical collections nestled within the garden's enchanting landscapes. Amongst the remarkable transformations, the revered camellias collection received special attention, as it underwent a comprehensive rejuvenation process under his skilful guidance. The recent publication of a global digital dataset of Camellia names provides the opportunity to publish the dataset of Camellia species, hybrids and its cultivars currently cultivated at Terra Nostra Garden with their validated names.

**New information:**

In June 2023, a total of 669 *Camellia* phenotypes were identified across the 12.5 hectares of Terra Nostra Garden. These phenotypes include 38 species, 178 hybrids and 637 cultivars. *Camelliajaponica* represents 81.7% of the 459 species cultivars, while *C. x williamsii* accounts for 32% of 178 hybrid cultivars. The most prevalent genotypes in *Camellia* hybrids with known parentage are those of *C.japonica*, *C.saluenensis* and *C.reticulata* present respectively in 64.1%, 45.5% and 37.9% of the hybrids. Regarding cultivar registration, 46.9% were registered in the US, followed by 13% from Japan and 10.8% from Portugal. Although the most ancient cultivar registered growing at the garden is *Camelliareticulata* Lindl. 'Damanao' from 1621, the majority (69.4%) of cultivars in the garden were registered in the 20^th^ century, followed by the 19^th^ century cultivars (20.7%). One cultivar, *Camellia* 'Patrícia Bensaude Fernandes', was produced and registered specifically by this garden.

## Introduction

Camellia gardens are important for their horticultural value (Kay et al. 2011), cultural significance (Xin et al. 2015), tourism and recreational opportunities (Chen and Sun 2018), conservation and research efforts (Blackmore et al. 2011, Mounce et al. 2017) and environmental benefits (Primack and Miller-Rushing 2009). They are cherished spaces that celebrate the beauty and significance of Camellia species, while contributing to various aspects of human well-being (Waylen 2006) and ecological balance (Donaldson 2009). Therefore, gardens of exotic plants offer a therapeutic and immersive experience (Hsieh et al. 2021), connecting individuals with nature (Oh et al. 2022), stimulating creativity, providing educational opportunities, promoting physical exercise and recreation and raising awareness about environmental conservation. Gardens of native plants are also highly relevant for educational puposes, as well as for research and native species conservation (Bishop et al. 2016).

In Azorean Islands, there are very important urban gardens with cultural and aesthetic importance (e.g. Jardim António Borges; Jardim Duque da Terceira, Jardim José do Canto, Jardim do Palácio de Sant'Ana, Terra Nostra Garden), but also an urban garden of native plants (Botanical Garden of Faial Island) (Arteaga et al. 2020). These urban gardens provide not only spaces for relaxation and enjoyment, but also play a significant role in preserving the region's botanical heritage, showcasing local and international plant species and contributing to the overall beauty and cultural fabric of the islands (Sousa 2000, Albergaria et al. 2021).

**Figure 1.**
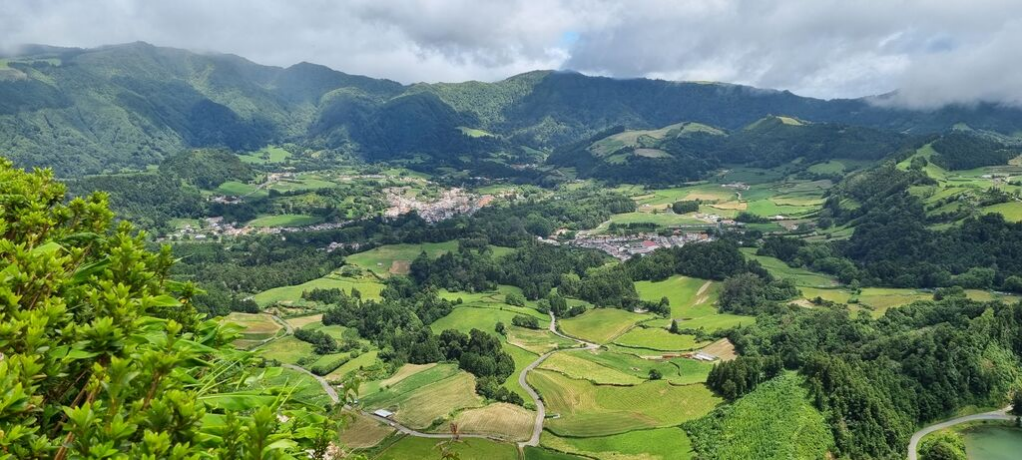
Caldera of Furnas active stratovolcano (Credit: Carina Costa).

Of particular relevance is the Terra Nostra Garden (Carvalho 2022). Nested inside the caldera of Furnas active stratovolcano, in Furnas Village, São Miguel Island (Azores Archipelago, Portugal) (Fig. 1), this garden has more than two centuries of history, an impressive landscape architecture and a range of botanical collections that are of global importance. One of the highlights of the garden is undoubtedly the Camellias Collection, which was awarded with the prestigious title of 'Camellia Garden of Excellence' by the International Camellia Society in 2014 (Costa et al. 2023b).

Terra Nostra Garden has seen a significant boost in tourism in the last two decades, with an impressive 268,044 entries in 2022. This influx of visitors has provided ample opportunity for maintenance, recovery and innovation efforts to be carried out within the garden. Today, the continued garden’s improvement requires a management approach, based on digital technology, comprehensive information about the various specimens, their locations and the necessary maintenance actions essential for maintaining the beauty and integrity of Terra Nostra Garden (Costa et al. 2023b).

In this comprehensive contribution, our primary objective is to provide a detailed list of all Camellia species, hybrids and cultivars presently cultivated at Terra Nostra Garden, located in São Miguel, Azores, Portugal. This marks the initial step of an ambitious project dedicated to cataloguing the entire flora within the urban gardens of São Miguel Island.

## General description

### Purpose

In this paper, we present the dataset of Camellia species, hybrids and cultivars currently cultivated at Terra Nostra Garden, along with their internationally accepted names according to the DICR (Wang et al. 2021, Wang 2023). Additionally, we provide a concise description of the collection.

### Additional information

Camellia cultivars with pink or red flowers exist in solid-coloured flowers and in variegated flowers. Some cultivars with variegated flowers are of genetic origin (e.g. ‘Tama-no-ura’) (Tateishi et al. 2008). In other cases, this results from plant virus infections (*Camelliajaponica* L. 'Hagoromo') (Terada et al. 2020). Consequently, not all the Camellia phenotypes correspond to different genotypes. Thus, we counted the total number of different phenotypes.

## Project description

### Title

Terra Nostra Garden 2023 dataset of Camellia species, hybrids and cultivars

### Personnel

Carina Costa, Fernando Costa, António Trota, Paulo Monjardino, Maria J. Trota, Paulo A.V. Borges

### Study area description

**Figure 2.**
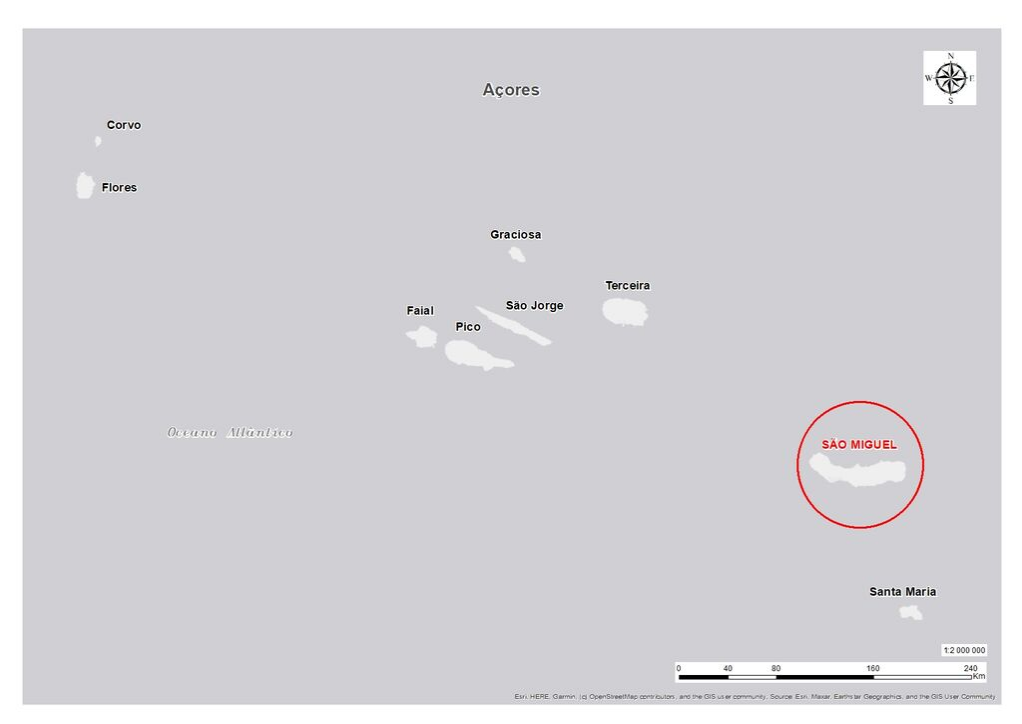
The map of Azores with the location of São Miguel Island.

**Figure 3.**
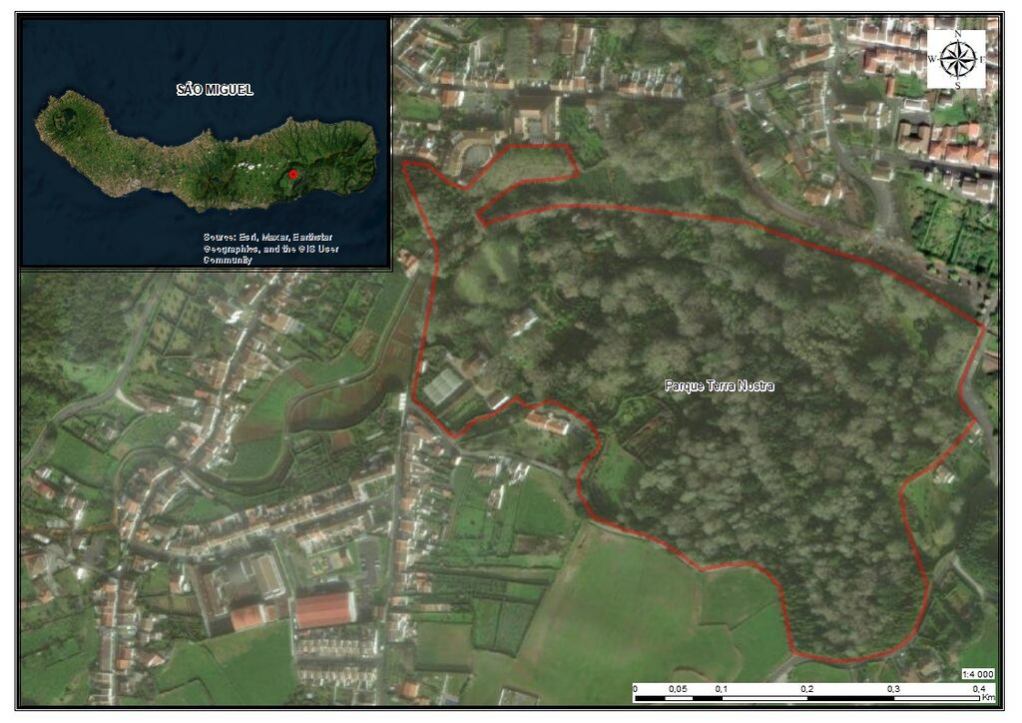
The location of Terra Nostra Garden in the area of Furnas in São Miguel Island.

In 1933, the 12 hectares property was acquired from several owners (Thomas Hickling, Thomas Hickling Jr., Viscount Duarte Borges da Câmara Medeiros, Marquis António Borges Medeiros Dias da Câmara e Sousa, Marquises' heirs) by the Terra Nostra Society, led by Vasco Bensaude. The current Terra Nostra Garden Hotel was inaugurated in 1935 and, two years later, the Terra Nostra Society reopened the botanical garden attached to the hotel, now called Terra Nostra Garden. The Terra Nostra Garden is located in S. Miguel Island (Azores, Portugal) (Fig. 2), nestled within the captivating surroundings of the Furnas volcano (Fig. 3).

### Design description

The georeferencing of garden areas was made in UTM coordinates, using official local reference coordinate system (PTRA08). The survey was possible after the installation of a permanent dense network of aesthetic topographic markers.

### Funding

Research was conducted within the scope of three research Centres: Centre of Biotechnology of the Azores (financed by FCT – Fundação para a Ciência e a Tecnologia, I.P., under the projects UIDP/05292/2020 and UIDB/05292/2020), Centre of Geosciences (under the project FCT-UIDB/50019/2020-2024) and the Centre for Ecology, Evolution and Environmental Changes/Azorean Biodiversity Group (financed by FCT – Fundação para a Ciência e a Tecnologia, I.P., under the project FCT-UIDB/00329/2020-2024 - Thematic Line 1 – integrated ecological assessment of environmental change on biodiversity and Azores DRCT Pluriannual Funding, Project M1.1.A/FUNC.UI&D/010/2021-2024).

## Sampling methods

### Study extent

Between 2019 and 2023, a total of 676 Camellia specimens were tagged and located in the garden. Once they bloomed, photographs of the flowers were taken and their morphological characteristics were compared to descriptions of species, subspecies, varieties, hybrids and registered cultivars using various resources, such as literature (e.g. Macoboy (1998), Jiyin et al. (2005), Jiyin (2007), Trehane et al. (2007), Garrido (2014)), journals (e.g. International Camellia Journal; the Camellia Journal, U.S.A.; Notiziario Società Italiana della Camelia, Italy), databases (e.g. Anonymous (2023), POWO (2023), Wang (2023)) and a non-published list of introduced Camellias compiled over the past 40 years (Fernando Costa unpublished notes, 1982-2022). This list includes acquisitions of identified specimens from reputable horticultural companies (e.g. António Assunção, Flavius Nursery, Guimarães, Portugal), as well as specimens identifications made during the 1^st^, 2^nd^ and 4^th^ International Meetings of Old Camellias at São Miguel Island (Sampaio and Albergaria 2006, Sampaio and Albergaria 2007, Sampaio and Albergaria 2013).

### Sampling description

The sampling procedure followed the "Plant Species Prospection" Darwin Core approach using direct observations in 20 plots/sections, located within the Terra Nostra Garden. The codes and coordinates of the 20 plots/sections can be consulted in the GBIF event table in Costa et al. (2023a). From an initial list of 774 names, five names corresponded to synonyms; 16 names are not registered in the International Camellia Register; 20 phenotypes once cultivated were found dead; 64 species cultivars remain to localise; therefore, efforts will be made to identify them in the next blooming periods.

### Quality control

Species and hybrid scientific names here used, with their authorities, follow the database ‘Plants of the World Online’ (POWO 2023). Cultivars names follow the ‘Database of International Camellia Register’ (Wang 2023).

## Geographic coverage

### Description

Terra Nostra Garden, Furnas, São Miguel Island, Azores, Portugal.

### Coordinates

37.76582963431182 and 37.774785412131244 Latitude; -25.317306518554688 and -25.302886962890625 Longitude.

## Temporal coverage

### Notes

Start date of sampling on 10-01-2023 and end date on 28-04-2023.

## Usage licence

### Usage licence

Creative Commons Public Domain Waiver (CC-Zero)

## Data resources

### Data package title

Terra Nostra Garden 2023 dataset of *Camellia* species, hybrids and cultivars

### Resource link

http://ipt.gbif.pt/ipt/resource?r=camelias_terra_nostra

### Alternative identifiers

https://www.gbif.org/dataset/f6090c89-867a-4e6f-9ae3-9e083712c319

### Number of data sets

2

### Data set 1.

#### Data set name

Event Table

#### Data format

Darwin Core Archive

#### Character set

UTF-8

#### Download URL

http://ipt.gbif.pt/ipt/resource?r=camelias_terra_nostra

#### Data format version

1.7

#### Description

The dataset is available on the Global Biodiversity Information Facility platform, GBIF (Costa et al. 2023a). The event table dataset is organised following the Darwin Core Archive (DwCA) format and contains 20 records (eventID).

**Data set 1.** 

Column label	Column description
id	A unique number for each event.
eventID	An identifier for every single event and specific to the dataset (Island code_area code_month_year_sampling protocol).
samplingProtocol	The methods or protocols used during an event.
sampleSizeValue	A numeric value for a measurement of the size (time duration, length, area or volume) of a sample in a sampling event.
sampleSizeUnit	The unit of measurement of the size (time duration, length, area or volume) of a sample in a sampling event.
eventDate	The unit of measurement of the size (time duration, length, area or volume) of a sample in a sampling event.
year	Year of the event.
month	Month of the event.
day	Month of the event.
habitat	Description of the habitat in which the Event occurred (Garden).
locationID	An identifier for the set of location information (specific to the dataset).
islandGroup	Name of the archipelago of the sampling site (Azores).
island	Name of the island of the sampling site (São Miguel).
country	Name of the country of the sampling site (Portugal).
countryCode	The standard code for the country in which the Location occurs (PT).
stateProvince	An identifier for every single event and specific to the dataset (Azores).
municipality	Municipality of the sampling site (Furnas).
locality	Name of the locality (Terra Nostra Garden).
minimumElevationInMetres	The lower limit of the range of elevation (altitude, usually above sea level), in metres.
decimalLatitude	Geographic coordinate (Decimal degrees): sampling location Latitude.
decimalLongitude	Geographic coordinate (Decimal degrees): sampling location Longitude.
geodeticDatum	Spatial reference system (SRS) upon which the geographic coordinates given in decimalLatitude and decimalLongitude are based.
coordinateUncertaintyInMetres	Coordinates' uncertainty in metres to the site of the true sampling area.
coordinatePrecision	A decimal representation of the precision of the coordinates given in the decimalLatitude and decimalLongitude.
georeferenceSources	A map, gazetteer or other resource used to georeference the Location.

### Data set 2.

#### Data set name

Occurrence Table

#### Data format

Darwin Core Archive

#### Character set

UTF-8

#### Download URL

http://ipt.gbif.pt/ipt/resource?r=camelias_terra_nostra

#### Data format version

1.7

#### Description

The dataset is available on the Global Biodiversity Information Facility platform, GBIF (Costa et al. 2023a). The occurrence table dataset is organised following the Darwin Core Archive (DwCA) format and contains 676 records (occurrenceID)

**Data set 2.** 

Column label	Column description
id	A unique number for each specimen.
type	The type of the related resource.
licence	Information about rights held in and over the resource.
rightsHolder	A person or organisation owning or managing rights over the resource (Terra Nostra Garden).
institutionID	An identifier for the institution having custody of the object(s) or information referred to in the record.
collectionID	An identifier for the collection or dataset from which the record was derived.
institutionCode	The name in use by the institution having custody of the object(s) or information referred to in the record.
collectionCode	An identifier for the collection or dataset from which the record was derived.
datasetName	The name identifying the dataset from which the record was derived (Inventory of Terra Nostra Garden Plant Taxa).
basisOfRecord	The specific nature of the data record (Human Observation).
occurrenceID	An identifier built as a "Globally Unique IDentifier".
recordedBy	Names of people responsible for recording the original occurrence.
lifeStage	The age class or life stage of the Organism(s) at the time the Occurrence was recorded.
establishmentMeans	The process of establishment of the species in the location, using a controlled vocabulary: “Introduced assisted colonisation".
eventID	An identifier for every single event and specific to the dataset (Island code_area code_month_year_sampling protocol).
identifiedBy	Names of people who assigned the Taxon to the subject.
dateIdentified	The date on which the subject was determined as representing the Taxon.
scientificName	Full scientific name, with authorship and date information, if known. When identification to species level was not possible, then it is the name in the lowest level taxonomic rank that can be determined.
kingdom	Scientific name of the kingdom in which the taxon is classified (Plantae).
phylum	Scientific name of the phylum in which the taxon is classified (Magnoliophyta).
class	Scientific name of the class in which the taxon is classified (Magnoliopsida).
order	Scientific name of the order in which the taxon is classified (Ericales).
family	Scientific name of the family in which the taxon is classified. (Theaceae).
genus	Scientific name of the genus in which the taxon is classified (Camellia).
specificEpithet	The species epithet of the scientific name.
infraspecificEpithet	Name of the lowest or terminal infraspecific epithet of the scientific name.
cultivarEpithet	The cultivar epithet of the scientific name.
taxonRank	The taxonomic rank of the most specific name in the scientific name.
scientificNameAuthorship	The authorship information related to the scientific name.
namePublishedIn	A reference for the publication in which the Cultivars were originally established under the rules of the associated dwc:nomenclaturalCode.
namePublishedInYear	The year of publication of the Cultivar names.

## Additional information

A total of 669 *Camellia* phenotypes were identified across the 12.5 hectares of Terra Nostra Garden. These phenotypes include 38 species (Table 1), 459 species cultivars (Table 2), 178 hybrid cultivars (Table 3), totalling 637 cultivars. From the 38 species, a total of 15 are only represented either by varieties or cultivars (see details in Table 1).

**Figure 4.**
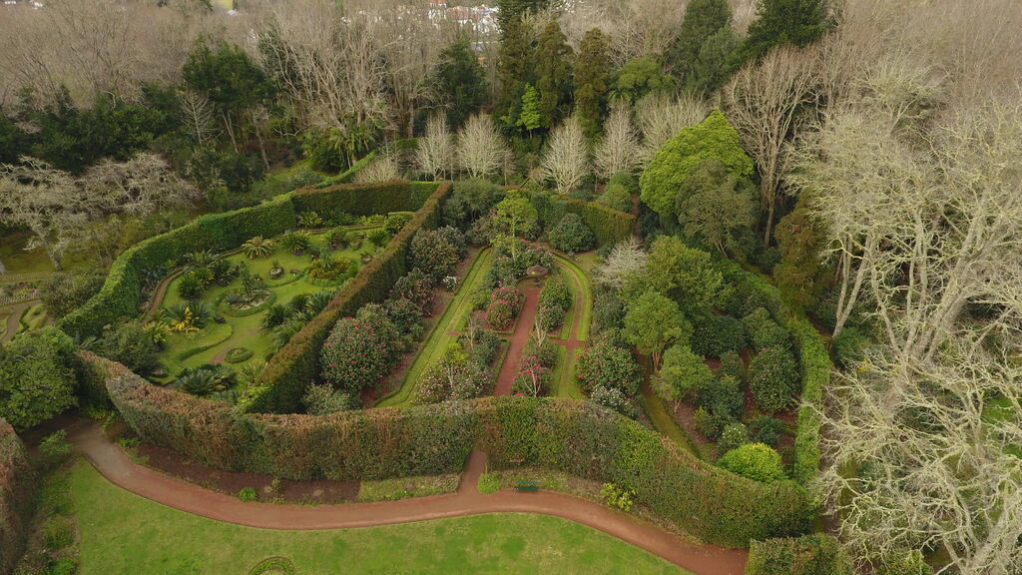
*Camelliareticulata* restricted area in the Terra Nostra Garden. Photo taken in March 2019 (Credit: Carina Costa).

*Camelliajaponica* represents 81.7% of the 459 species cultivars, while *C. x williamsii* accounts for 32% of 178 hybrid cultivars (Tables 2, 3). The most prevalent genotypes in *Camellia* hybrids with known parentage are those of *C.japonica*, *C.saluenensis* and *C.reticulata* (Fig. 4) present respectively in 64.1%, 45.5% and 37.9% of the hybrids (Table 4).

Regarding cultivar registration, 46.9% were registered in the US, followed by 13% from Japan and 10.8% from Portugal (Table 5). Although the most ancient cultivar registered growing at the garden is *Camelliareticulata* Lindl. 'Damanao' from 1621, the majority (69.4%) of cultivars in the garden were registered in the 20^th^ century, followed by the 19^th^ century cultivars (20.7%) (Table 6). One cultivar, *Camellia* 'Patrícia Bensaude Fernandes', was produced and registered specifically by this garden.

In this study, we listed 669 *Camellia* phenotypes that are available across the 12.5 hectares of Terra Nostra Garden. These phenotypes include 38 species, 178 hybrids and 637 cultivars. This collection can contribute to the preservation and conservation of worldwide *Camellia* plant diversity. Many *Camellia* species and varieties are endangered or threatened in their natural habitats, so maintaining a collection in a protected environment can help prevent their extinction. Moreover, this collection serves as an important educational resource, allowing visitors to learn about different Camellia species, their characteristics and growing requirements. In addition, Camellias are valued for their attractive and vibrant flowers and the Terra Nostra Garden collection is providing a visually appealing and relaxing environment for visitors to enjoy.

## Figures and Tables

**Table 1.** *Camellia* species present at Terra Nostra Garden in June 2023. Some species are not represented by the taxon rank species, but either by a variety or a cultivar.

**Camellia species**	**Represented by**
*Camelliaamplexicaulis* (Pit.) Cohen Stuart	
*Camelliaazalea*C.F.Wei	
*Camelliabrevistyla* (Hayata) Cohen Stuart	var. brevistyla
*Camelliachekiangoleosa* Hu	
*Camelliachrysanthoides* Hung T.Chang	
*Camelliacuspidata* (Kochs) Bean	var. cuspidata
*Camelliadrupifera* Lour.	
*Camelliaedithae* Hance	One cultivar
*Camelliaeuphlebia* Merr. ex Sealy	
*Camelliaflava* (Pit.) Sealy	
*Camelliaflavida* Hung T.Chang	var. flavida
*Camelliaforrestii* (Diels) Cohen Stuart	
*Camelliafraterna* Hance	
*Camelliagranthamiana* Sealy	
*Camelliagrijsii* Hance	var. grijsii
*Camelliahiemalis* Nakai	Six cultivars
*Camelliahongkongensis* Seem.	
*Camelliaimpressinervis* Hung T.Chang & S.Ye Liang	
*Camelliajaponica* L.	378 cultivars
*Camellialutchuensis* T.Itô ex T.Itô & Matsum.	var. lutchuensis
*Camelliamairei* (H.Lév.) Melch.	var. lapidea (Y.C.Wu) Sealy
*Camelliaoleifera*C.Abel	
*Camelliapetelotii* (Merr.) Sealy T.L.Ming & W.J.Zhang	var. petelotii
	var. microcarpa (S.L.Mo) T.L.Ming & W.J.Zhang
*Camelliapilosperma* S.Yun Liang	
*Camelliapitardii* Cohen-Stuart	One cultivar
*Camelliapolyodonta* F.C.How ex Hu	
*Camelliapubipetala* Y.Wan & S.Z.Huang	
*Camelliareticulata* Lindl.	30 cultivars
*Camelliarosiflora* Hook.	The species and one cultivar
*Camelliarosmannii* Ninh	
*Camelliarusticana* Honda	Seven cultivars
*Camelliasalicifolia* Champ. ex Benth.	
*Camelliasasanqua* Thunb.	39 cultivars
*Camelliasinensis* (L.) Kuntze	var. sinensis
*Camelliasynaptica* Sealy	
*Camelliataliensis* (W.W.Sm.) Melch.	
*Camelliatsaii* Hu	
*Camelliauraku* Kitam.	

**Table 2.** Number of Terra Nostra Garden *Camellia* cultivars (Cv) in 2023.

**Camellia species**	**Cv (n)**	**Cv (%)**
*C.japonica* L.	375	81.7
*C.sasanqua* Thunb.	39	8.5
*C.reticulata* Lindl.	29	6.3
*C.rusticana* Honda	7	1.5
*C.hiemalis* Nakai	6	1.3
*C.edithae* Hance	1	0.2
*C.pitardii* Cohen-Stuart	1	0.2
*C.rosiflora* Hook.	1	0.2
**Total species cultivars**	**459**	

**Table 3.** Number of Terra Nostra Garden *Camellia* hybrids (Hy Cv) in 2023.

**Camellia hybrids**	**Hy Cv (n)**	**Hy Cv (%)**
*C.japonica* x *C.saluenensis* (*C. x williamsii*)	57	32
*C.hybrids* (Unknown species parentage)	33	18.5
*C.reticulata* hybrids	23	12.9
*C.reticulata* x *C.japonica*	15	8.4
*C.japonica* x *C.sasanqua* (*C.* x *vernalis*)	8	4.5
*C.petelotii* hybrids	6	3.4
*C.japonica* x *C.petelotii*	5	2.8
*C.saluenensis* x *C.reticulata*	5	2.8
*C.japonica* x *C.reticulata*	4	2.2
*C.sasanqua* x *C.reticulata*	4	2.2
*C.reticulata* x *C.granthamiana*	2	1.1
*C.saluenensis* hybrids	2	1.1
*C.japonica* x *C.lutchuensis*	1	0.6
*C.lutchuensis* x *C.japonica*	1	0.6
*C.reticulata* x *C.sasanqua*	1	0.6
*C.reticulata* x *C.saluenensis*	1	0.6
*C.cuspidata* x *C.saluenensis*	1	0.6
*C.pitardii* x *C.japonica*	1	0.6
*C.rosiflora* x *C.tsaii*	1	0.6
*C.rusticana* x *C.lutchuensis*	1	0.6
*C.cuspidata* hybrid	1	0.6
*C.granthamiana* hybrid	1	0.6
*C.kissi* hybrid	1	0.6
*C.lutchuensis* hybrid	1	0.6
*C.pitardii* hybrid	1	0.6
*C.transnokoensis* hybrid	1	0.6
**Total hybrid cultivars**	**178**	

**Table 4.** Contribution of known species genotypes to the composition of hybrid Camellias. CJ = *C.japonica*; CSa = *C.saluenensis*; CRe= *C.reticulosa*; CSas = *C . sasanqua*; CPe = *C.petelotii*; CL = *C.lutchuensis*; CG = *C.granthamiana*; CC = *C.cuspidata*; CPi = *C.pitardii*; CK = *C.kissi*; CRo = *C.rosiflora*; CRu = *C.rusticana*; CTr = *C.transnokoensis*; CTs = *C.tsaii*.

		**Camellia genotypes**													
**Camellia hybrids**	**n**	**CJ**	**CSa**	**CRe**	**CSas**	**CPe**	**CL**	**CG**	**CC**	**CPi**	**CK**	**CRo**	**CRu**	**CTr**	**CTs**
*C.japonica* x *C.saluenensis* (C. x williamsii)	57	57	57												
*C.reticulata* hybrids	23			23											
*C.reticulata* x *C.japonica*	15	15		15											
*C.japonica* x *C.sasanqua*	8	8			8										
*C.* x *vernalis*		8			8										
*C.petelotii* hybrids	6					6									
*C.japonica* x *C.petelotii*	5	5				5									
*C.saluenensis* x *C.reticulata*	5		5	5											
*C.japonica* x *C.reticulata*	4	4		4											
*C.sasanqua* x *C.reticulata*	4			4	4										
*C.reticulata* x *C.granthamiana*	2			2				2							
*C.saluenensis* hybrids	2		2												
*C.japonica* x *C.lutchuensis*	1	1					1								
*C.lutchuensis* x *C.japonica*	1	1					1								
*C.reticulata* x *C.sasanqua*	1			1	1										
*C.reticulata* x *C.saluenensis*	1	1	1	1											
*C.cuspidata* x *C.saluenensis*	1		1						1						
*C.pitardii* x *C.japonica*	1	1								1					
*C.rosiflora* x *C.tsaii*	1											1			1
*C.rusticana* x *C.lutchuensis*	1						1						1		
*C.cuspidata* hybrid	1								1						
*C.granthamiana* hybrid	1							1							
*C.kissi* hybrid	1										1				
*C.lutchuensis* hybrid	1						1								
*C.pitardii* hybrid	1									1					
*C.transnokoensis* hybrid	1													1	
**n**	**145**	**93**	**66**	**55**	**13**	**11**	**4**	**3**	**2**	**2**	**1**	**1**	**1**	**1**	**1**
%	**100**	**64.1**	**45.5**	**37.9**	**9**	**7.6**	**2.8**	**2.1**	**1.4**	**1.4**	**0.7**	**0.7**	**0.7**	**0.7**	**0.7**

**Table 5.** Number of cultivars released per country.

**Country**	**N**	%
United States	299	46.9
Japan	83	13.0
Portugal	69	10.8
New Zealand	43	6.8
Australia	34	5.3
Italy	29	4.6
United Kingdom	25	3.9
China	26	4.1
France	15	2.4
Belgium	11	1.7
Spain	3	0.5
**Sum**	**637**	**100**

**Table 6.** Number of cultivars released per century.

**Century**	**N**	%
17^th^	4	0.6
18^th^	8	1.3
19^th^	132	20.7
20^th^	442	69.4
21^st^	51	8.0
**Sum**	**637**	**100**
